# Upregulation of lactate dehydrogenase a by 14-3-3ζ leads to increased glycolysis critical for breast cancer initiation and progression

**DOI:** 10.18632/oncotarget.9136

**Published:** 2016-05-02

**Authors:** Chia-Chi Chang, Chenyu Zhang, Qingling Zhang, Ozgur Sahin, Hai Wang, Jia Xu, Yi Xiao, Jian Zhang, Sumaiyah K. Rehman, Ping Li, Mien-Chie Hung, Fariba Behbod, Dihua Yu

**Affiliations:** ^1^ Department of Molecular and Cellular Oncology, The University of Texas MD Anderson Cancer Center, Houston, TX 77030, USA; ^2^ Cancer Biology Program, Graduate School of Biomedical Sciences at Houston, Houston, TX 77030, USA; ^3^ Department of Pathology and Laboratory Medicine, The University of Kansas Medical Center, Kansas City, KS 66160, USA

**Keywords:** LDHA, 14-3-3ζ, glycolysis, cancer metabolism, breast cancer initiation

## Abstract

Metabolic reprogramming is a hallmark of cancer. Elevated glycolysis in cancer cells switches the cellular metabolic flux to produce more biological building blocks, thereby sustaining rapid proliferation. Recently, new evidence has emerged that metabolic dysregulation may occur at early-stages of neoplasia and critically contribute to cancer initiation. Here, our bioinformatics analysis of microarray data from early-stages breast neoplastic lesions revealed that 14-3-3ζ expression is strongly correlated with the expression of canonical glycolytic genes, particularly lactate dehydrogenase A (LDHA). Experimentally, increasing 14-3-3ζ expression in human mammary epithelial cells (hMECs) up-regulated LDHA expression, elevated glycolytic activity, and promoted early transformation. Knockdown of LDHA in the 14-3-3ζ-overexpressing hMECs significantly reduced glycolytic activity and inhibited transformation. Mechanistically, 14-3-3ζ overexpression activates the MEK-ERK-CREB axis, which subsequently up-regulates LDHA. *In vivo*, inhibiting the activated the MEK/ERK pathway in 14-3-3ζ-overexpressing hMEC-derived MCF10DCIS.COM lesions led to effective inhibition of tumor growth. Therefore, targeting the MEK/ERK pathway could be an effective strategy for intervention of 14-3-3ζ-overexpressing early breast lesions. Together, our data demonstrate that overexpression of 14-3-3ζ in early stage pre-cancerous breast epithelial cells may trigger an elevated glycolysis and transcriptionally up-regulating LDHA, thereby contributes to human breast cancer initiation.

## INTRODUCTION

One in five breast cancer patients presents early-stage disease in the clinic, such as atypical ductal hyperplasia (ADH) and/or ductal carcinoma *in situ* (DCIS) [1]. Although advances in targeted therapies have substantially reduced the mortality rate for breast cancer patients [2], a better mechanistic understanding of cancer initiation will undoubtedly lead to significant improvement in disease prevention and further reduce breast cancer-specific mortality.

Breast cancer initiation and progression involve multiple cellular alterations, including metabolic dysregulation [3–5]. Recently, new evidence has emerged to suggest that metabolic perturbation, including glycolytic shift, starts at early-stage diseases that contribute to the early transformation of normal epithelial cells and the initiation of cancer [6, 7]. The Warburg effect is a metabolic abnormality of cancer cells characteristic of elevated aerobic glycolysis, which promotes production of biological building blocks for cell proliferation [8]. Critical mediators of the Warburg effect include transcription factors *MYC* and *HIF-1α* [9]. However, it remains unclear what other mediators may promote Warburg effect, especially during the early-stage breast cancer initiation and progression.

14-3-3ζ belongs to a family of scaffold proteins that are involved in many important cellular functions and regulate multiple signal transduction pathways [10, 11]. For example, 14-3-3ζ overexpression enhances AKT activation by forming a complex with PI3K in the cell membrane [12], accelerates cell proliferation via miR221-mediated p27 downregulation [13], turns off TGF-β's cytostatic function [11], and reduces apoptosis through direct binding with FOXO3a in breast cancer cells [1]. Interestingly, overexpression of 14-3-3ζ occurs at ADH early-stage breast disease [1, 14].

Given the concomitant 14-3-3ζ overexpression and abnormal metabolic alterations during the early-stage breast disease, we postulated that overexpression of 14-3-3ζ may lead to increased glycolysis and contribute to early transformation of mammary epithelial cells and subsequent breast cancer initiation/progression. To this end, we examined gene expression profiling data of early-stage neoplastic breast lesions and found a strong correlation between the expressions of 14-3-3ζ and genes of the canonical glycolytic pathway, particularly lactate dehydrogenase A (LDHA). By exogenously overexpressing 14-3-3ζ in nontransformed human mammary epithelial cells (hMECs), we identified a direct mechanistic link between 14-3-3ζ overexpression and LDHA upregulation. We revealed that 14-3-3ζ-mediated LDHA upregulation is critical to early transformation of hMECs. Our data provide direct evidence that 14-3-3ζ overexpression in early-stage breast disease contributes critically to the metabolic dysregulation of hMECs, and that 14-3-3ζ confers a metabolic advantage to initiate neoplastic transformation. Importantly, our data also demonstrate that targeting 14-3-3ζ-induced metabolic dysregulation could be an efficacious strategy for prevention and early intervention of early-stage breast cancer.

## RESULTS

### 14-3-3ζ overexpression increases glycolysis in human breast epithelial cells

**Figure 1:**
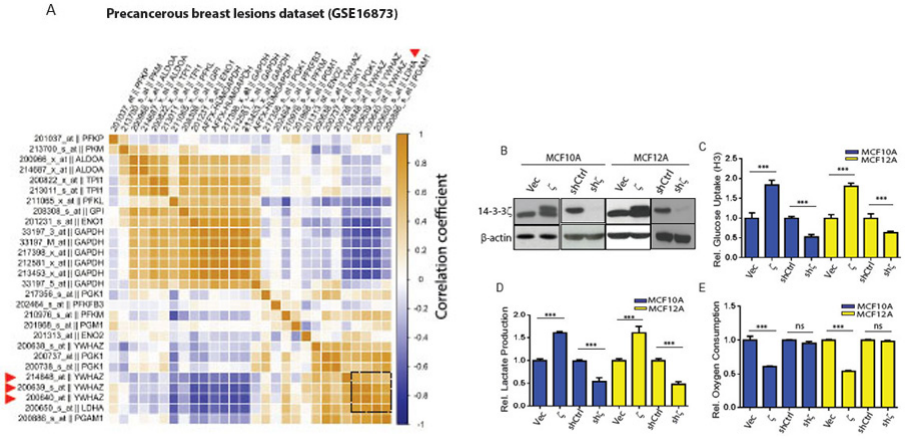
The 14-3-3ζ expression level correlates with glycolytic genes in early-stage breast cancer patients and its overexpression increases glycolysis in hMECs **A.** Heat map of pairwise correlation on 14-3-3ζ (YWHAZ) and glycolytic gene (Gene Ontology, GO:0006096) expression levels in the early-stage breast lesions (GSE16873). Triangles indicate the high correlation between 14-3-3ζ and LDHA. **B.** western blotting for 14-3-3ζ protein expression in 14-3-3ζ overexpressing and 14-3-3ζ-knockdown MCF10A and MCF12A cells, compared to control cells. C-E, MCF10A and MCF12A cells with 14-3-3ζ-overexpression (10A.ζ and 12A.ζ) and 14-3-3ζ-knockdown (10A.shζ and 12A.shζ) were assessed for glucose uptake **C.** lactate production **D.** and oxygen consumption **E.** The relative levels of glucose uptake, lactate production, and oxygen consumption of the 10A.ζ cells and 12A.ζ cells were normalized to those of the control 10A.Vec and 12A.Vec cells, respectively; and the relative levels of glucose uptake, lactate production, and oxygen consumption of the 10A.shζ cells and 12A.shζ cells were normalized to those of the control 10A.shCtrl and 12A.shCtrl cells, respectively. Absolute measurements were normalized to the corresponding controls. Bars indicate standard deviations. ***, *P*<0.001; n.s., not significant by the Student t-test.

Since metabolic dysregulations has recently been implicated to take place during the early-stage of neoplastic transformation [6, 7], and abnormal 14-3-3ζ overexpression was also observed in pre-cancerous breast lesions [14], we hypothesized that 14-3-3ζ overexpression contributes to metabolic dysregulations in early-stage breast cancer. To gain insights on this conjecture, we first examined the relationship between 14-3-3ζ expression and genes involved in metabolic functions, using a microarray dataset (GSE16873) generated from histologically normal epithelia, simple ductal hyperplasia (SDH), atypical ductal hyperplasia (ADH) and ductal carcinoma in situ (DCIS) [15]. Remarkably, the 14-3-3ζ expression level is most strongly correlated with the expressions of genes involved in glycolysis (Gene Ontology, GO:0006096) in these pre-cancerous and early-stage breast lesions (Figure 1A). In addition, this strong correlation between 14-3-3ζ expression level and glycolytic genes persisted in breast cancer patients [16] (Supplementary Figure S1, GSE2109).

To test whether 14-3-3ζ functionally modulates cellular glycolytic activity in pre-cancerous mammary epithelial cells, we measured glycolytic activities in widely used and validated models of pre-cancerous mammary epithelial cells, i.e., MCF10A and MCF12A hMECs. We compared three glycolytic indices (i.e., glucose uptake, lactate production, and oxygen consumption) in MCF10A and MCF12A hMECs that had either exogenous 14-3-3ζ overexpression (10A.ζ and 12A.ζ cells) or endogenous 14-3-3ζ knockdown (10A.shζ, and 12A.shζ cells) (Figure 1B) [11]. Indeed, glucose uptake and lactate production significantly increased in both 10A.ζ and 12A.ζ cells compared to this control cells, but significantly reduced in both 10A.shζ, and 12A.shζ cells compared to their control cells (Figure 1C and 1D). Cells with higher glycolysis activity tend to reduce the rates of oxidative phosphorylation and oxygen consumption and shift the metabolic flux from the ATP-generating TCA cycle to the biomass-producing glycolysis. Consistently, the oxygen consumption rates of the 14-3-3ζ-overexpressing 10A.ζ and 12A.ζ cells were significantly reduced than that of the control cells (Figure 1E). However, no significant difference in oxygen consumption was detected between the 10A.shζ and 12A.shζ cells and their control cells (Figure 1E), suggesting that 14-3-3ζ does not modulate basal oxygen consumption in the 14-3-3ζ low-expressing MCF10A and MCF12A cells. Next, we evaluated the overall impact of 14-3-3ζ on cellular glycolytic activity by calculating the glycolytic index, G x L / O, where G is for glucose uptake rate, L is for lactate generation production, and O is for oxygen consumption rate [17]. 14-3-3ζ-overexpressing 10A.ζ and 12A.ζ cells had 4 to 5 fold increases, whereas 14-3-3ζ knockdown 10A.shζ and 12A.shζ cells had approximately 70% decreases, in their glycolytic index compared to their respective control cells (Supplementary Table S1 and S2). Collectively, these data demonstrate that 14-3-3ζ positively modulates glycolytic activity in nontransformed MCF10A and MCF12A cells.

### 14-3-3ζ overexpression upregulates LDHA leading to increased aerobic glycolysis

**Figure 2:**
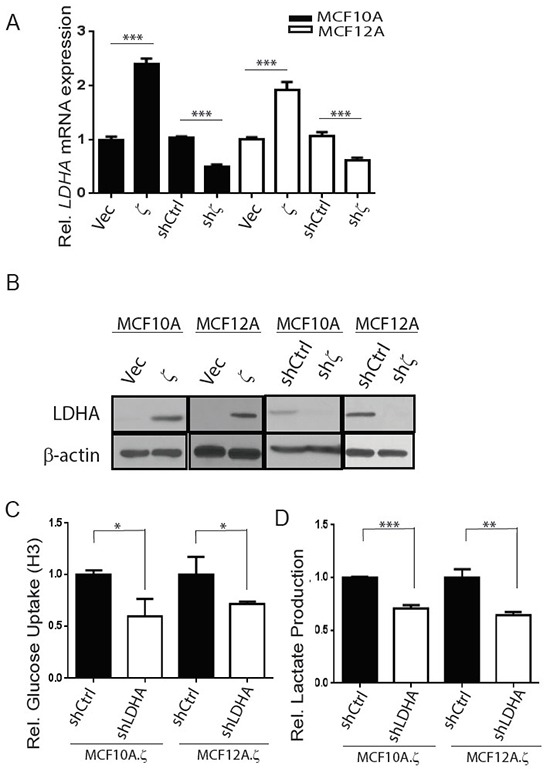
14-3-3ζ overexpression increases glycolysis by upregulating LDHA mRNA and protein expressions in hMECs **A–B.** qRT-PCR analysis of relative *LDHA* mRNA expression (A), western blotting for LDHA protein expression (B) in 14-3-3ζ overexpressing (10A.ζ and 12A.ζ) and 14-3-3ζ-knockdown (10A.shζ and 12A.shζ) MCF10A and MCF12A cells, compared to control cells. The film was exposed for 30 seconds to detect LDHA protein expression in 14-3-3ζ-overexpressiong cells and their vector control; and the film was exposed for 3 minutes to detect LDHA protein expression in 14-3-3ζ-knockdown cells and their shCtrl cells in both MCF10A and MCF12A cells. C-D, 14-3-3ζ overexpressing MCF10A and MCF12A cell lines with LDHA knockdown (10A.ζ.shLDHA and 12A.ζ.shLDHA) were assessed for glucose uptake **C.** and lactate production **D.** The relative levels of glucose uptake and lactate production in the 10A.ζ.shLDHA and 12A.ζ.shLDHA cells were normalized to those of the 10A.ζ.shCtrl and 12A.ζ.shCtrl cells, respectively. Bars indicate standard deviations. *, *P*<0.05; **, *P*<0.01; ***, *P*<0.001 using the Student t-test.

To investigate the molecular mechanisms of the 14-3-3ζ-mediated increase of glycolysis, we focused on LDHA because its expression, compared with that of other glycolytic genes in human pre-cancerous lesions, is more strongly associated with 14-3-3ζ expression level (Figure 1A). Furthermore, 14-3-3ζ-overexpressing 10A.ζ and 12A.ζ cells had significantly increased levels of LDHA mRNA and protein expression compared to their vector control cells; whereas knockdown of 14-3-3ζ in 10A.shζ and 12A.shζ cells led to significantly decreased LDHA mRNA and protein levels compared to the control shCtrl cells (Figure 2A and 2B).

These findings led us to investigate whether 14-3-3ζ-mediated LDHA upregulation directly contributes to the increase of glycolytic activity. We knocked down the LDHA gene expression in the 14-3-3ζ overexpressing 10A.ζ and 12A.ζ cells by shRNA (10A.ζ.shLDHA and 12A.ζ.shLDHA cells) and measured the three glycolytic indices. The 10A.ζ.shLDHA and 12A.ζ.shLDHA cells had significantly less glucose uptake and lactate production than the control cells (Figure 2C and 2D); whereas oxygen consumption in 10A.ζ.shLDHA and 12A.ζ.shLDHA cells was not significantly different from that in the control cells (data not shown). Overall, the glycolytic indices were reduced with LDHA knockdown by approximately 70% (10A.ζ.shLDHA versus 10A.ζ.shCtrl cells) and 60% (12A.ζ.shLDHA versus 12A.ζ.shCtrl cells) (Supplementary Table S3). Together, these data indicate that 14-3-3ζ-mediated LDHA upregulation is a key promoting factor of glycolysis in 14-3-3ζ overexpressing hMECs.

### 14-3-3ζ-mediated LDHA upregulation contributes to early-stage transformation of hMECs

**Figure 3:**
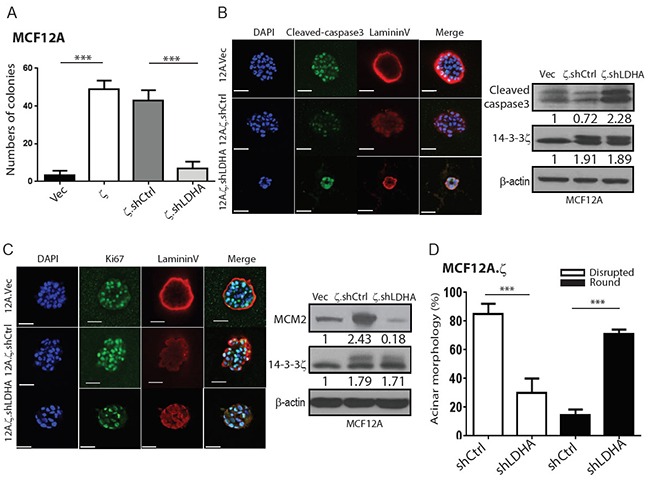
14-3-3ζ-mediated LDHA upregulation contributes to the early-stage transformation of hMECs **A.** Quantified soft colony formation assay of the MCF12A sublines. 14-3-3ζ overexpressing MCF12A (12A.ζ) cells, 12A.ζ cells with LDHA knockdown (12A.ζ.shLDHA), and the corresponding control cells were cultured in 0.5% soft agar for 21 days, and the colonies (≥ 50μm diameter) were counted. **B.** Detection of apoptosis (cleaved caspase 3, green), polarity markers (Laminin V, red) and DAPI (blue) in MCF12A sublines cultured in Matrigel for 8 days. Left: Representative images of immunofluorescence staining. Scale bars, 50 μm. Right: Western blotting of cleaved caspase-3 and 14-3-3ζ in acini lysate collected from 3D culture. **C.** Detection of proliferation marker (Ki-67, green), polarity marker (Laminin V, red) and DAPI (blue) in MCF12A sublines cultured in 3D culture for 15 days. Left: Representative images of immunofluorescence staining. Scale bars, 50 μm. Right: Western blotting of MCM2, and 14-3-3ζ in acini collected from 3D culture. **D.** MCF12A sublines in 3D culture for 20 days, rounded or disrupted acinar morphology were counted.

As increased glycolysis has been implicated in early-stage neoplastic transformation [8], we examined whether 14-3-3ζ-mediated LDHA upregulation is a critical determinant of hMEC transformation. We previously reported that 14-3-3ζ overexpression in MCF10A cells (10A.ζ) led to increased colony formation in soft agar and dysregulated 3D acini [1, 14], which mimics early transformation of mammary gland *in vivo*. Consistent with our previous findings in MCF10A cells, the colony numbers of 14-3-3ζ-overexpressing MCF12A cells (12A.ζ) were also significantly increased compared to vector control (12A.Vec) cells (Figure 3A). Remarkably, LDHA knockdown in 14-3-3ζ-overexpressing MCF10A (10A.ζ.shLDHA) and MCF12A (12A.ζ.shLDHA) cells reversed the transforming effects of 14-3-3ζ overexpression, yielding significantly fewer colonies compared to their control cells (Figure 3A and Supplementary Figure S2A).

Furthermore, in the 3D culture system, 14-3-3ζ-overexpressing 12A.ζ cells also developed dysregulated acini with filled lumen resulting from reduced apoptosis (Figure 3B) and increased proliferation [18] (Figure 3C) compared to control 12A.Vec cells. Notably, LDHA knockdown in 14-3-3ζ high expressing 10A.ζ and 12A.ζ (10A.ζ.shLDHA and 12A.ζ.shLDHA) partially reversed the dysregulated acini phenotype with increased cleaved-caspase 3 (Figure 3B, and Supplementary Figure S2B), suggesting that LDHA has an anti-apoptosis effect in hMECs. In addition, 12A.ζ.shLDHA cells had reduced proliferation (reduced both Ki67 and MCM2 levels) but 10A.ζ.shLDHA showed a mild reduction in proliferation (Figure 3C and Supplementary Figure S2C). Further analysis revealed that the 10A.ζ.shLDHA and 12A.ζ.shLDHA cells had fewer disruptive acini structures and more rounding acini than their control cells (Figure 3D and Supplementary Figure S2D), demonstrating a partial restoration of normal epithelial morphology. Collectively, these data demonstrate that 14-3-3ζ overexpression-mediated LDHA upregulation contributes, at least partially, to hMEC transformation and aberrant acinar formation through, increased glycolysis, increased proliferation, and decreased apoptosis that can be reversed by LDHA downregulation.

### 14-3-3ζ overexpression leads to transcriptional up-regulation of LDHA

Since LDHA upregulation contributes to 14-3-3ζ-mediated hMEC transformation, it is important to identify the underlying molecular mechanisms of 14-3-3ζ-induced LDHA upregulation. Because 14-3-3ζ overexpression increases LDHA expression at both mRNA and protein levels in hMECs (Figure 2A and 2B) and is known to regulate protein stability [19, 20], we compared the LDHA protein stability of the 10A.ζ and 12A.ζ cells with that of their control 10A.Vec and 12A.Vec cells, but found no significant difference between them (Supplementary Figure S3A and S3B). This led us to focus on studying mechanisms of *LDHA* mRNA upregulation by 14-3-3ζ. We examined *LDHA* mRNA stability by treating 10A.ζ and 10A.Vec cells with actinomycin D, and measured *LDHA* mRNA degradation rate. No significant difference in *LDHA* mRNA stability was found between the 10A.ζ and 10A.Vec cells (Supplementary Figure S3C), suggesting that 14-3-3ζ might modulate *LDHA* mRNA expression through transcriptional regulation.

**Figure 4:**
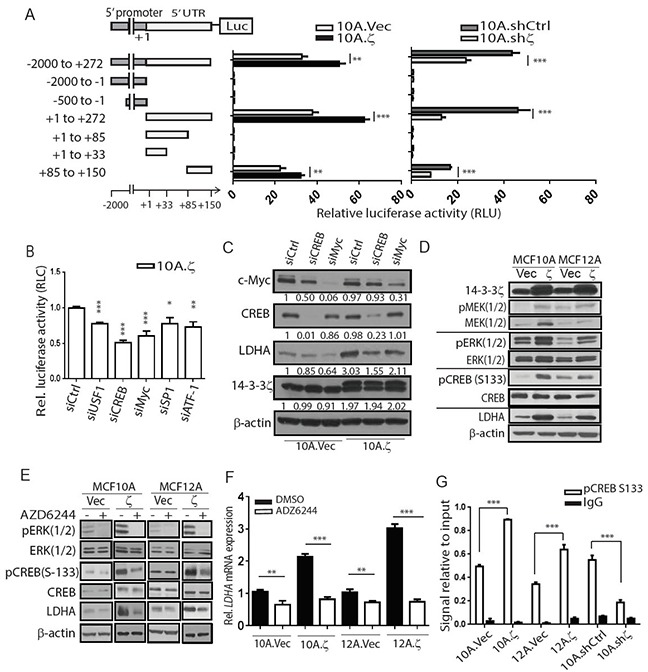
14-3-3ζ overexpression transcriptionally upregulates LDHA **A.** Schematic design of the LDHA regulatory region deletion assay. Series of deletion constructs were transfected into 10A.ζ, 10A.Vec, 10A.shζ and 10A.shCtrl cells. **B.** Cells with individual knockdown of five indicated transcription factors were subject to luciferase activity assays after transfection with the construct with the 65bp 5′UTR region driving the luciferase reporter gene. The relative luciferase activity of the cells was normalized to that of 10A.ζ cells with control siRNA. **C.** Western blotting of c-Myc, CREB, LDHA, and 14-3-3ζ in MCF10A sublines. **D.** Western blotting of phospho-MEK1/2, phospho-ERK, phospho-CREB, total MEK1/2, ERK, CREB, LDHA, and 14-3-3ζ in the indicated hMEC sublines. **E.** Western blotting detecting the phospho-ERK, phospho-CREB, total ERK, CREB, LDHA, and 14-3-3ζ in10A.Vec, 10A.ζ, 12A.Vec and 12A.ζ cells treated with AZD6244 or DMSO. **F.** qRT-PCR analysis of *LDHA* mRNA expression in the hMEC sublines following by treatment with AZD6244 or DMSO. **G.** Chromatin immunoprecipitation assay using anti-phospho-CREB (Ser-133) followed by PCR (ChIP-PCR) of the LDHA promoter region. Bars indicate standard deviations. *, *P*<0.05; **, *P*<0.01; ***, *P*<0.001 by the Student t-test.

To determine whether 14-3-3ζ transcriptionally upregulates *LDHA*, we cloned the LDHA promoter from 2000 base pairs (bp) upstream of the transcription start site plus the entire 272bp 5′-untranslated region (5′-UTR) into the pGL3-Basic vector to drive luciferase reporter gene expression (−2000 to +272-Luc), and transfected this reporter vector into the 10A.ζ versus 10A.Vec cells and the 10A.shζ versus 10A.shCtrl cells (Figure 4A). We detected an increase of luciferase activity in 10A.ζ compared to 10A.Vec cells, but a decreased luciferase activity in 10A.shζ compared to 10A.shCtrl cells, indicating that 14-3-3ζ transcriptionally upregulates *LDHA* mRNA expression (Figure 4A).

To identify the cis-regulatory element in the −2000bp LDHA promoter and 5′-UTR region that is responsible for 14-3-3ζ-induced transcriptional upregulation, we made a series of deletion constructs in the −2000 to +272 region, subcloned them into the pGL3-Basic vector, and transfected them into the 10A.ζ, 10A.Vec, 10A.shζ, and 10A.shCtrl cells (Figure 4A, left). Remarkably, a specific 65bp region (+85 to +150) in the 5′-UTR of LDHA gene was necessary and sufficient to induce the luciferase gene expression (Figure 4A). Transfection of this 65bp 5′-UTR region resulted in higher luciferase activity in 10A.ζ than in vector control cells (Figure 4A, middle). In contrast, the luciferase activity driven by the 65bp 5′-UTR was lower in the 10A.shζ cells compared to the 10A.shCtrl cells (Figure 4A, right).

Next, to identify the transcription factors that bind to the 65bp 5′-UTR region of LDHA and are responsible for transcriptional upregulation by 14-3-3ζ, we analyzed this DNA sequence for putative transcription factor binding sites using the Transcription Element Search System (TESS) and University of California-Santa Cruz (UCSC) genome browser online analysis tools [21, 22]. We identified potential binding sites for five transcription factors USF1, CREB, MYC, SP1, and ATF-1 (Supplementary Figure S4A), knocked down them individually in the 10A.ζ cells and compared their luciferase activities. Knocking down each of the five genes reduced the LDHA 65bp 5′-UTR driven luciferase activity in 10A.ζ cells to various degrees and knocking down CREB led to the most significant reduction (Figure 4B).

Furthermore, we examined *LDHA* mRNA and protein levels in the 10A.Vec and 10A.ζ cells that had the five transcription factors knocked down individually. Among these five transcription factors, CREB and MYC knock down in the 10A.ζ cells (Supplementary Figure S4B and S4C), significantly reduced *LDHA* protein and mRNA compared to shCtrl cells (Figure 4C and Supplementary Figure S4D). However, CREB knockdown had less effect on the LDHA protein level in the control 10A.Vec cells compared to MYC knockdown cells (Figure 4C), suggesting that CREB has a more specific role in regulating LDHA expression in 14-3-3ζ overexpressing cells. Therefore, we focused on how 14-3-3ζ overexpression leads to CREB transactivation of LDHA that contributes to early transformation in 14-3-3ζ overexpressing hMECs.

14-3-3ζ has been shown to directly bind to Raf and activate MEK/ERK [23–25], which directly phosphorylate p90 ribosomal S6 kinase, that in turn activates and phosphorylates of CREB at Ser-133 [26, 27]. Thus, we tested whether CREB-mediated transcriptional upregulation of LDHA could be modulated through the 14-3-3ζ-ERK-RSK signaling axis. Indeed, we detected a dramatically higher activation of the ERK pathway and increased phosphorylation of CREB at Ser-133 in both 10A.ζ and 12A.ζ cells compared to control 10A.Vec and 12A.Vec hMECs (Figure 4D). Furthermore, treating the 14-3-3ζ-overexpressing 10A.ζ and 12A.ζ cells with a potent MEK/ERK inhibitor, AZD6244, dramatically inhibited the ERK pathway, reduced CREB phosphorylation and decreased the LDHA mRNA and protein expressions to similar levels as in the 10A.Vec and 12A.Vec hMECs (Figure 4E and 4F), suggesting that LDHA upregulation in the 14-3-3ζ overexpressing 10A.ζ and 12A.ζ cells is primarily dependent on the ERK/CREB pathway. To determine whether the transcription factor CREB directly binds to the *LDHA* promoter, we performed chromatin immunoprecipitation assay using anti-p-Ser-133-CREB followed by LDHA promoter-specific PCR (ChIP-PCR). We found significantly more *LDHA* promoter-bound pCREB proteins (~1.8 to 2 fold) in the 14-3-3ζ-overexpressing 10A.ζ and 12A.ζ cells than in the control cells (Figure 4G). In contrast, knockdown of 14-3-3ζ in MCF10A hMECs significantly reduced pCREB protein binding to the LDHA promoter compared to the 10A.shCtrl cells (Figure 4G). Together, these data indicate that 14-3-3ζ overexpression activates the MEK/ERK pathway and consequently increases the binding of pCREB to the LDHA promoter, leading to LDHA transcriptional upregulation.

### Targeting MEK/ERK/CREB pathway effectively inhibits LDHA expression and tumor outgrowth in a 14-3-3ζ overexpressing DCIS model

As the above data demonstrated that the MEK/ERK/CREB pathway is critical for 14-3-3ζ-induced LDHA upregulation which contributes to hMEC early transformation. Conceivably, targeting this pathway to inhibit metabolic adaptation of early-stage breast cancer cells towards glycolysis may be an effective strategy to intervene cancer progression. Therefore, we next investigated whether targeting the 14-3-3ζ downstream MEK/ERK pathway may effectively prevent or intervene the early-stage breast cancer further progression *in vivo.* To this end, we exogenously overexpressed 14-3-3ζ in early-stage DCIS model of MCF10DCIS.COM cells. The MCF10DCIS.COM line is a MCF10A cells-derived model that forms DCIS-like mammary lesions and ultimately progresses to invasive mammary tumors in nude mice [28]. Recent studies revealed almost identical genomic profiles between the MCF10A and MCF10DCIS.COM cells, supporting our efforts to extend the above studies of MCF10A to in vivo investigations using the MCF10DCIS.COM line [29]. Consistent with the MCF10A and MCF12A cells, exogenous overexpression of 14-3-3ζ in the MCF10DCIS.COM cells (DCIS.COM.ζ) led to activation of the ERK/CREB pathway and LDHA upregulation *in vivo*; whereas 14-3-3ζ knockdown in MCF10DCIS.COM led to decreased ERK/CREB activity and LDHA downregulation (Supplementary Figure S5 and S6).

**Figure 5:**
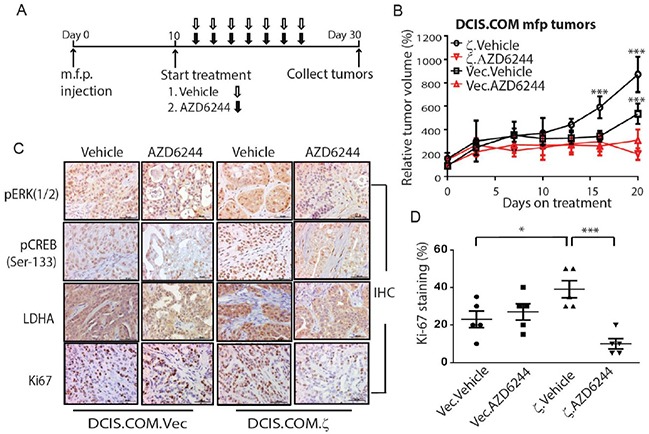
AZD6244 treatment inhibits DCIS COM.ζ tumor growth. **A.** Experimental design of treating the DCIS.COM.Vec and DCIS.COM.ζ m.f.p. tumors in nude mice. After DCIS.COM.Vec and DCIS.COM.ζ xenograft tumors were established by day 10, mice were treated with vehicle or AZD6244 daily. **B.** The relative tumor volume of DCIS.COM.Vec.Vehicle and DCIS.COM.ζ.Vehicle were compared with their AZD6244 treatment groups, respectively. Bars indicate standard deviations. **C.** Representative images of IHC staining of phospho-ERK, phospho-CREB (Ser-133), LDHA and Ki-67 in DCIS.COM.Vec and DCIS.COM.ζ xenograft tumors from mice treated with vehicle and AZD6244. Scale bars, 50 μm. **D.** Quantitative analysis of Ki-67-positive cells in DCIS.COM.Vec and DCIS.COM.ζ xenograft tumors. *, *P*<0.05; ***, *P*<0.001 by the Student t-test.

We implanted DCIS.COM.ζ and control DCIS.COM.Vec cells into the mammary fat pads (mfps) of nude mice (day 0) to produce tumor xenografts and started treating these mice with vehicle or MEK/ERK inhibitor AZD6244 on day 10 (Figure 5A). Treatment continued until day 30, when the mice were sacrificed and their mammary tumors were collected. vehicle-treated 14-3-3ζ overexpressing DCIS.COM.ζ (DCIS.COM.ζ.Vehicle) tumors had significantly higher tumor growth rate compared to vehicle-treated DCIS.COM.Vec (DCIS.COM.Vec.Vehicle) tumors (Figure 5B), suggesting that overexpression of 14-3-3ζ can promote tumor outgrowth. Interestingly, AZD6244 inhibited >75% DCIS.COM.ζ tumor growth but <50% DCIS.COM.Vec tumor growth, suggesting that the 14-3-3ζ overexpressing DCIS.COM.ζ tumors were more dependent on the MEK/ERK pathway and its downstream targets. These data indicate that 14-3-3ζ overexpression-mediated tumor progression in this DCIS model can be effectively targeted by MEK/ERK inhibitor.

To determine the impact of MEK/ERK inhibitor treatment on 14-3-3ζ/ERK/CREB/ LDHA, as well as on tumor cell proliferation and apoptosis, we collected tumors from control and treatment groups. IHC analysis showed that 14-3-3ζ overexpressing DCIS.COM.ζ tumors had increased phospho-ERK and phospho-CREB levels that correlated with higher LDHA expression compared with DCIS.COM.Vec tumors (Figure 5C) but had no significant effect on total ERK, CREB or 14-3-3ζ expression levels (Supplementary Figure S6). AZD6244 treatment significantly reduced phospho-ERK, phospho-CREB and LDHA levels in DCIS.COM.ζ and DCIS.COM.Vec tumors compared to vehicle treatment (Figure 5C and Supplementary Table S4). Compared to the DCIS.COM.Vec.Vehicle tumors, the DCIS.COM.ζ.Vehicle tumors showed no significant difference in apoptosis, but a singnificantly increased Ki67 positive proliferating cells (Figure 5D), which were inhibited by AZD6244 (Figure 5C and 5D). These data indicate that AZD6244 effectively inhibit the MEK/ERK/CREB/LDHA axis and proliferation of 14-3-3ζ overexpressiong tumor cells, thereby suppressing DCIS-like tumor outgrowth.

### The 14-3-3ζ-LDHA axis as potential biomarkers for predicting clinical outcome

**Figure 6:**
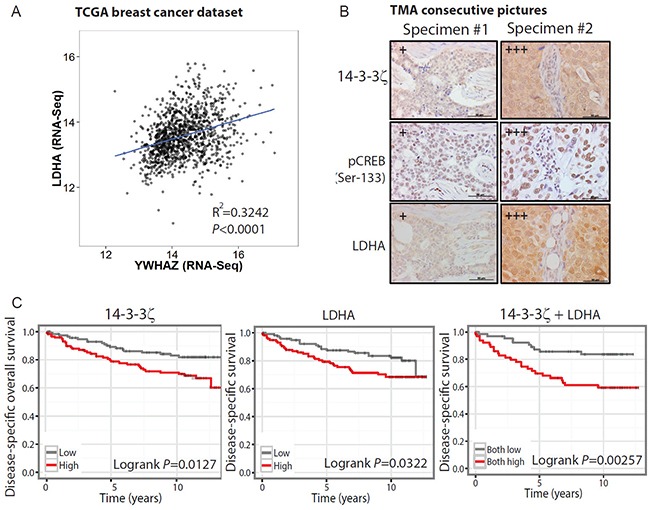
The 14-3-3ζ-LDHA signaling axis holds prognostic value in predicting clinical outcome **A.** Linear regression of the LDHA and 14-3-3ζ (YWHAZ) expression values in the TCGA breast cancer dataset. The square of Pearson coefficient R^2^ is 0.324 and *P*<0.0001. **B.** Representative images of IHC staining of 14-3-3ζ, phospho-CREB (Ser-133) and LDHA, in a 208-core human breast cancer tissue microarray (TMA). A total of 187 (89%) cores were eligible for scoring and examined for the association of 14-3-3ζ and phosphor-CREB; a total of 183 (88%) cores were examined the association of 14-3-3ζ and LDHA. The IHC staining scores for 14-3-3ζ, p-CREB, LDHA were defined as 0, 1+, 2+, or 3+, where 3+ indicates higher positive expression; and 1+ indicates lower positive expression. Scale bars, 50 μm. **C.** Kaplan-Meier survival curve of breast cancer patients (GSE3494). Concomitant high expression of 14-3-3ζ and LDHA (logrank *P*<0.01) predicts worse outcome than high 14-3-3ζ (YWHAZ) expression level (logrank *P*<0.05) or high LDHA expression level (logrank *P*<0.05).

Having established that the 14-3-3ζ/MEK/ERK/CREB/LDHA axis is potently active in nontransformed hMECs and highly correlated in early neoplastic breast lesions (R^2^>0.8) (Figure 1A). Next, we extended our examination by bioinformatics analysis of datasets generated from breast cancers. We found that the correlative relationship between the expression levels of 14-3-3ζ (YWHAZ) and LDHA reached R^2^ of 0.32 and 0.31 (Figure 6A and Supplementary Figure S1) in RNAseq-derived TCGA dataset [30], and microarray-derived dataset GSE2109[16] respectively. The strength of such positive correlation is much weaker than that in early-stage diseases (Figure 1A), suggesting that with cancer progression into advanced stages, neoplastic cells may have metabolitically adapted to a complex tumor environment. Furthermore, when we examined LDHA levels in 14-3-3ζ overexpressing established breast cancer cell lines such as, HCC1954 HER2+ and MDA-MB-231 TNBC, we did not detect a significant up-regulation of LDHA or increase of glycolysis (data not shown). These data suggest that the 14-3-3ζ/CREB/LDHA pathway may be more critical in the early-stage breast cancer initiation.

As the above bioinformatics analyses were performed on RNA data, we further evaluated the clinical relevance of 14-3-3ζ/CREB/LDHA axis at protein level using tissue microarray (TMA) of mixed stages of breast cancers. Indeed, IHC staining revealed that the 14-3-3ζ protein levels were significantly correlated with the LDHA protein levels in these breast cancer specimens (Figure 6B and Supplementary Table S5). Importantly, the 14-3-3ζ expression levels were also significantly correlated with CREB phosphorylation in the consecutive TMA slides from the same group of patients (Figure 6B and Supplementary Table S5).

We then evaluated whether 14-3-3ζ and LDHA levels hold prognostic values for breast cancer. As there is no early-lesion dataset with follow-up on clinical outcome, we instead used a breast cancer gene expression dataset (GSE3494) with disease-specific overall survival data. We found that concomitant high expression of 14-3-3ζ and LDHA predicts worse survival of breast cancer patients compared to high expression of either gene alone (*P*=0.00257 vs. *P*=0.0127 and *P*=0.0322) (Figure 6C). Furthermore, the 5-year survival rate for patients with concurrent high expression of both 14-3-3ζ and LDHA genes dropped almost 10% compared to high expression of either gene alone (Figure 6C). These data suggest that when combined together, the expression levels of 14-3-3ζ and LDHA have a more power in predicting the clinical outcome of breast cancer patients. It is possible that 14-3-3ζ and LDHA expression levels together may have an even stronger power in predicting the clinical outcome of early-stage disease progression in patients, which should be investigated in future studies.

## DISCUSSION

**Figure 7:**
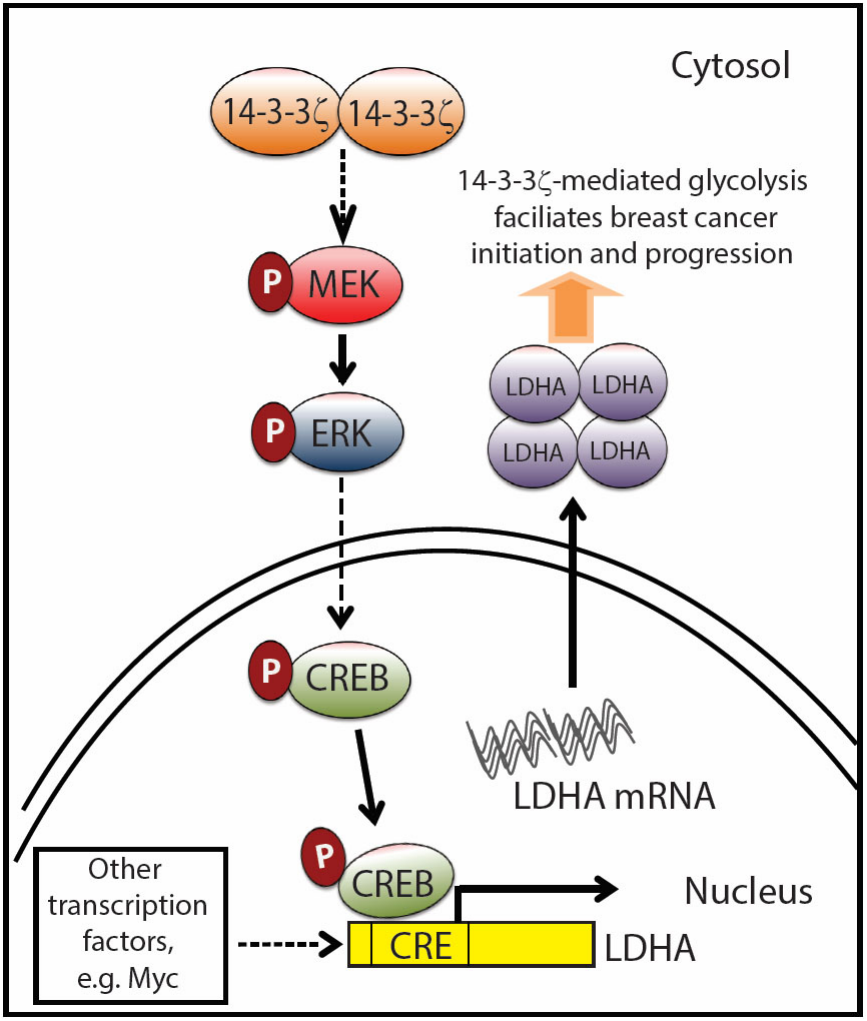
Proposed model of 14-3-3ζ overexpression-mediated LDHA upregulation facilitates breast cancer tumorigenesis Schema showing that 14-3-3ζ overexpression activates the MEK/ERK/CREB pathway and transcriptionally upregulates LDHA which contributes to increasing glycolysis and promotes cancer initiation and growth. Dotted arrow points out that other transcription factors including Myc can regulate LDHA gene expression [38], reprogram cancer metabolism and contribute to cancer pathogenesis as well. 14-3-3ζ-mediated LDHA upregulation shows the novel functional role of 14-3-3ζ in mediating metabolic dysregulation in cancer.

Increased glycolysis in cancer cells is a general phenomenon to satisfy the demanding needs of rapid cell proliferation. However, recent studies suggests that such metabolic dysregulation may start at very early stage of epithelial cell transformation [6, 7] and can be used for early cancer detection and diagnosis [31]. In this study, we identified that 14-3-3ζ overexpression-mediated LDHA upregulation contributed to the metabolic switch toward glycolysis and cell transformation in early-stage breast cancer. In addition to our previous studies showing that exogenous 14-3-3ζ overexpression cooperates with ErbB2 to promote DCIS to IDC transition [32]. Here, we demonstrated a novel role of 14-3-3ζ in mediating early-stage breast cancer progression by upregulating LDHA expression and cellular glycolysis (Figure 7).

In early-stage neoplastic lesions, the expression of 14-3-3ζ strongly and positively associated with the expression of LDHA. However, when the disease progressed into advanced stages, the correlation between 14-3-3ζ and LDHA is not very strong. It suggests that 14-3-3ζ-induced LDHA upregulation is critical for hMEC early transformation and breast cancer early initiation but may be less critical as cancer progresses and adapts to a more complex metabolic environment. Nevertheless, 14-3-3ζ/LDHA axis still provides prognostic values for overall survival of breast cancer patients (Figure 6C). LDHA is known to be regulated by other oncogenes such as *HIF-1a* during cancer progression, thus cancer cells have expanded regulatory mechanism to confer metabolic advantages in the advanced stages of diseases. Previous studies showed that 14-3-3ζ is involved in multiple functions including chemoresistance, epigenetics regulation, adipogenesis and metastasis [11, 33–35]. Here, we demonstrated a novel role of 14-3-3ζ in cancer cell metabolism and our findings bring new insights into the mechanistic understanding of 14-3-3ζ overexpression-mediated breast cancer initiation and progression of early-stage breast diseases.

LDHA is a CREB target gene because the LDHA promoter has *c-AMP* response element (CRE) [36]. A genome-wide promoter analysis of CREB target genes showed that many of the conserved CREs (TGACGTCA) exist either within 1000-bp of the ATG initiation codon or within 250-bp of the 5′-UTR sequence in mouse and human genome [36]. In our study, 10A.ζ transfected with the 65bp region of the 5′-UTR showed higher luciferase activity than those with the 2000-bp upstream promoter region (Figure 4A), suggesting that the CRE binding site in the 5′-UTR is critical for CREB binding and transactivation of *LDHA* mRNA in hMECs. CREB activation is mediated through phosphorylation by multiple upstream pathways [37]. Here, we demonstrated that 14-3-3ζ overexpression upregulates LDHA expression and increases glycolysis through the MEK/ERK/CREB signaling axis in early breast cancer, delineating the important signaling pathway for an essential cellular phenotype. Although previous studies showed that c-Myc is critical to regulate LDHA expression and promote cancer progression [38, 39], however, in our models, CREB is more important for LDHA upregulation in 14-3-3ζ-overexpressing hMECs (Figure 4C). Additionally, we demonstrated strong correlation between 14-3-3ζ, CREB phosphorylation and LDHA expression levels in human breast cancers by IHC staining of patient-derived tissue microarray (TMA), which extended our *in vitro* and preclinical findings to human breast cancer specimens and validated the clinical relevance of our studies.

Because metabolic dysregulation is a hallmark of cancer [40, 41], targeting abnormally altered metabolic pathways in cancer cells has emerged as an attractive therapeutic approach that is being actively investigated [42, 43]. In highly proliferating cells and cancer cells, LDHA is a key enzyme in the glycolytic cascade that converts pyruvate to lactate and cycles NADH back to NAD^+^ to sustain rapid proliferation [44, 45]. After identifying the important roles of the 14-3-3ζ/ERK/CREB/LDHA signaling axis in mediating cellular glycolysis, we showed that targeting MEK/ERK pathway in 14-3-3ζ overexpressing DCIS.COM tumors (DCIS.COM.ζ) with AZD6244 significantly inhibited the mammary tumor growth by inhibiting proliferation. Although MEK/ERK could potentially modulate multiple downstream targets, our data clearly demonstrated that inhibiting MEK/ERK leads to reduced LDHA and tumor inhibition. These data suggest that 14-3-3ζ-mediated LDHA upregulation and metabolic dysregulation could be intervened concurrent by targeting for MEK/ERK and early intervention of cancer [46].

In summary, our studies defined a novel role of 14-3-3ζ during early transformation and early-stage breast cancer by transcriptionally upregulating LDHA and functionally increasing glycolysis, which ultimately facilitate breast cancer initiation and progression. Furthermore, our data in a preclinical DCIS model indicated that inhibiting the MEK/ERK pathway could be an effective strategy for intervention of the early-stage breast cancer, and this strategy may potentially be applicable in the clinic.

## MATERALS AND METHODS

### Cell lines and cell culture

MCF10A and MCF12A cells were obtained from ATCC and were maintained in hMECs medium [11]. MCF10DCIS.COM cells, provided by Dr. Fariba Behbod [28], were maintained in DMEM/F-12 media supplemented with 5% horse serum. All cell lines were authenticated and validated by MD Anderson Cancer Center's Characterized Cell Line Core.

### Plasmids and shRNAs

MCF10A, MCF12A and MCF10DCIS.COM cell lines were transfected with pcDNA3-HA-14-3-3ζ or pcDNA3 empty vector and selected with neomycin for 14-3-3ζ-overexpressing sublines. 14-3-3ζ shRNA (clone, NM_003406.2-418s1c1) and LDHA shRNA (NM_005566) were used to make 14-3-3ζ and LDHA knockdown cell lines.

### Bioinformatics

Glycolysis-related genes were extracted from the Gene Ontology database under the term “glycolytic process” (accession #GO:0006096). The corresponding patient-derived gene expression values were retrieved from the Gene Expression Omnibus data repository microarray datasets for early-stage breast neoplasia (GSE16873) and advanced breast cancers (GSE2109), as well as the RNA-seq datasets from the TCGA data portal. The clustering analysis of gene expression and heatmap visualization of the correlation matrix were performed in the corrplot package (v0.73) on the statistical computation platform R (v3.1.2). Breast cancer patient-derived gene expression dataset GSE3494 had clinical follow-up information and was used to investigate the relationship between 14-3-3ζ and/or LDHA expression and patient prognosis. Patients were stratified as having either “low” or “high” expression of the gene by median expression values of 14-3-3ζ and LDHA in the data cohort, respectively. Patients with “low” or “high” expression of both 14-3-3ζ and LDHA genes were further extracted to investigate whether there is synergistic predictive effect to combine the two biomarkers together. The Kaplan-Meier plots and survival analysis of breast cancer patients (GSE3494) were performed using R package survival (v2.38).

### Real-time PCR analyses

Total RNA was extracted with Trizol reagent following manufacturer's protocol. *LDHA, CREB, MYC* mRNA expression were determined by qRT-PCR using TaqMan primers (Hs00855332_g1, Hs00231713_m1 and Hs00153408_m1) and normalized to 18S rRNA endogenous control (#4310893E).

### Metabolic assays

For glucose uptake assays, cells were starved for 3 hours before 2-deoxy-D-glucose-[1,2-^3^H(N)] (Moravek Biochemicals) was added in. Tritium signal was measured by liquid scintillation counting. For lactate production assay, cells were plated for 24 hours, followed by fresh-medium incubation for 1 hour. Lactate production was measured according to the manufacturer's instructions (BioVision Inc). For oxygen consumption assays, cells were plated in a 96-well oxygen biosensor system (BD Biosciences) and according to the manufacturer's instructions [47]. The overall cellular glycolytic activity was evaluated as previously described [17].

### Soft agar colony formation assay

Anchorage-independent cell growth was analyzed by the methods previously describe [14].

### Luciferase reporter assay

The LDHA promoter fragments were amplified from a bacterial artificial chromosome (#RP11-107C21) and cloned into a pGL3-basic vector. The promoter constructs were co-transfected with a *Renilla* luciferase control reporter vector into cells using the Amaxa HMEC Nucleofector kit, program T-24 (Lonza). Bioluminescence activity was assessed by methods previously described [11].

### Chromatin immunoprecipitation (ChIP) assay

ChIP assays were performed following previously described methods [48]. Immunoprecipitation antibodies: phospho-CREB and IgG antibodies. Primers for qPCR: Fwd 5′-TGGCTCGGCATCCAC-3′ and Rev 5′- CTGCAGCACTCTGAGCTG-3′.

### siRNAs and chemical inhibitors

siRNA control and siRNAs were purchased from Sigma for LDHA, CREB, MYC, ATF1, USF1, and SP1 knockdown *in vitro*. Cells were transfected with siRNAs using Pepmute siRNA Transfection Reagent (SignaGen Laboratories) according to manufacturer's protocol. The final siRNA concentration was 25nM for each transfection. AZD6244 (Selumetinib) was obtained from Selleck Chemicals and AdooQ Bioscience (#A10257). AZD6244 (10μM) was used to treat cells *in vitro* for at least 5 hours prior to lysate collection from cell culture.

### Three-dimensional culture and immunofluorescence staining

All of the three-dimensional cultureand immunofluorescence procedures were using methods described previously [49].

### Tumor xenograft studies

All the mouse experiments were carried out in accordance with protocols approved by MD Anderson's Institutional Animal Care Committee. We established breast cancer xenografts by injecting 5×10^5^ MCFDCIS.COM.Vec or MCFDCIS.COM.ζ cells orthotopically into the mammary fat pads of 6-week-old female SWISS^nu/nu^ mice. We then divided the mice randomly into two groups, AZD6244 treatment or vehicle control group. Each group contained 5 mice. AZD6244 was suspended in sterile HPMC solution (0.5 % HPMC, 0.1 % Tween 80 in 25 mM citrate) and given to mice through oral gavage daily at a dose of 16 mg/kg body weight or with vehicle control. Tumor growth was monitored with caliper measurements every 4 days for 3 weeks. Tumor volume was calculated using the formula volume = length x (width)^2^/2 [50]. Mice were sacrificed 20 days after treatment initiation and tumors were collected and embedded in paraffin following a routine pathological procedure.

### Immunohistochemistry (IHC) analyses and tissue microarray (TMA)

Antigen retrieval and IHC analyses were performed as described previously [32]. We used the following antibodies: anti-14-3-3ζ (Santa Cruz); anti-LDHA, anti-CREB, anti-phospho-CREB, anti-ERK, anti-phospho-ERK, anti-cleaved caspase 3 (Cell Signaling); and anti-MCM (Epitomics). For TMA, we used a 70-case, 208-core breast cancer tissue microarray (catalog no. BR208, US Biomax Inc.).

### Statistical analyses

Between-group differences were assessed using the Student's t test or ANOVA. *P* values < 0.05 were considered statistically significant.

## SUPPLEMENTARY FIGURES AND TABLES


