# Supplementary material for: Upregulation of lactate dehydrogenase a by 14-3-3ζ leads to increased glycolysis critical for breast cancer initiation and progression
Source: Oncotarget. 2016 May 2;7(23):35270–83. doi: 10.18632/oncotarget.9136 (PMC5085227; doi:10.18632/oncotarget.9136)
Supplement: Supplementary file 1 [file oncotarget-07-35270-s001.pdf]

SUPPLEMENTARY FIGURES AND TABLES

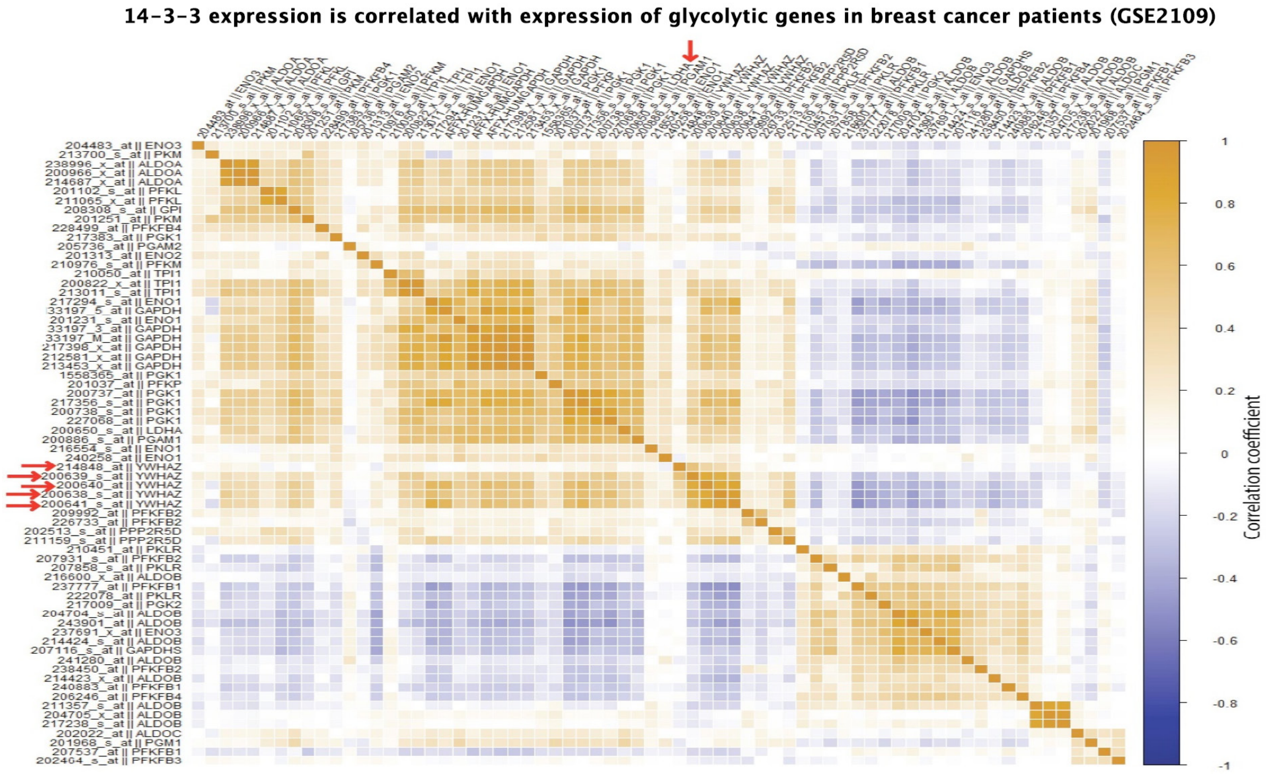

Supplementary Figure S1: Heat map of pairwise correlation on 14-3-3ζ and glycolytic gene expression levels in breast cancer patients profiled with cDNA microarray (expO, GSE2109). The datasets contain data from 353 breast cancer patients. Five 14-3-3ζ (YWHAZ) probe sets were analyzed together with glycolysis-related genes (GO: 0006096). Probe sets were ordered according to clustering analysis. Arrow indicates the strong correlation between 14-3-3ζ and LDHA.

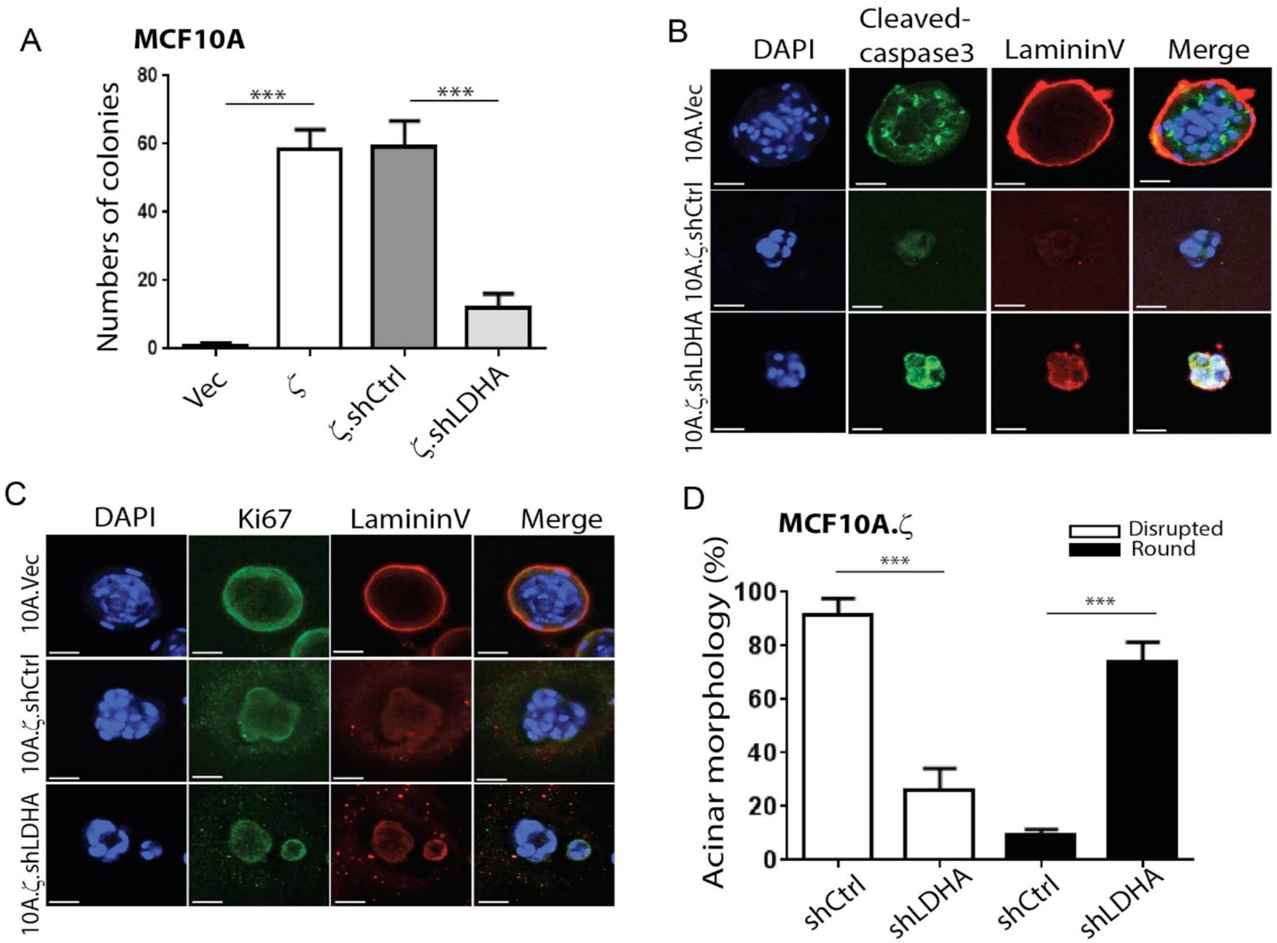

Supplementary Figure S2: 14-3-3ζ-mediated LDHA upregulation contributes to early-stage transformation of MCF10A cells (related to Figure 3). A. Quantified soft colony formation assay of the MCF10A sublines. 14-3-3ζ-overexpressing MCF10A (10A.ζ) cells and 10A.ζ cells with LDHA knockdown (10A.ζ.shLDHA) were cultured in 0.5% soft agar for 45 days, and the cell colonies were counted. **B.** Detection of apoptosis (cleaved caspase-3, green) and polarity marker (laminin V, red) and DAPI (blue) in MCF10A sublines cultured in Matrigel. Representative images of immunofluorescence staining. Scale bars, 20 μm. **C.** Detection of proliferation markers (Ki-67, green), polarity marker (laminin V, red), and DAPI (blue) in MCF10A sublines cultured in Matrigel. Representative images of MCF10A sublines subjected to immunofluorescence staining. **D.** MCF10A sublines cultured for 30 days were assessed for rounded or disrupted acinar morphology.

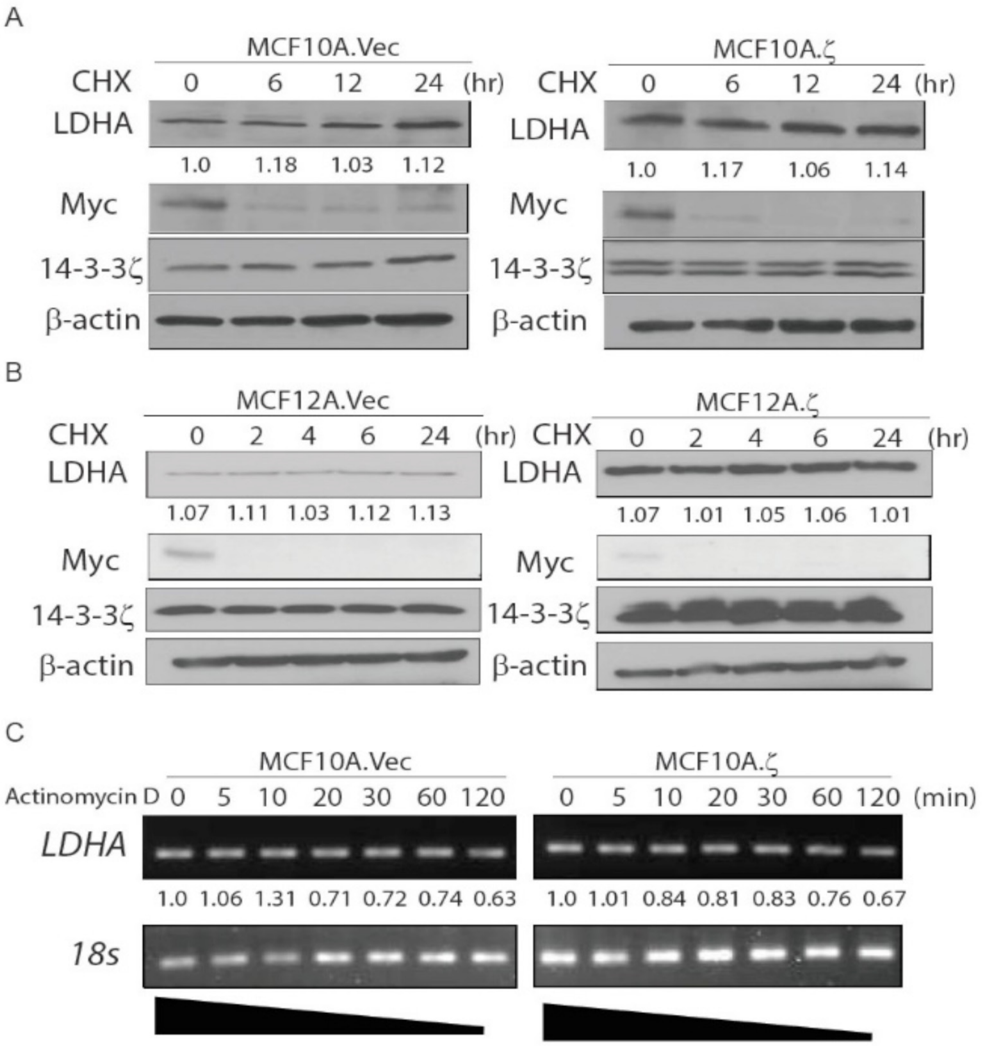

Supplementary Figure S3: A–B. MCF10A and MCF12A sublines were treated with 100 µg/mL cycloheximide (CHX) from 0 to 24 hours and compared the LDHA degradation rate over the time. Myc served as a positive control to show that CHX worked effectively during treatment. The LDHA expression levels were normalized to β-actin and also to their time zero of treatment. **C.** MCF10A sublines were treated with actinomycin D from 0 to 2 hours and compared the mRNA level of *LDHA* over the time. After 2 hours treatment, both 14-3-3ζ-overexpressing cells and vector control cells showed that they have similar degradation rate. The *LDHA* mRNA expression levels were normalized to *18s* and also to their time zero of treatment.

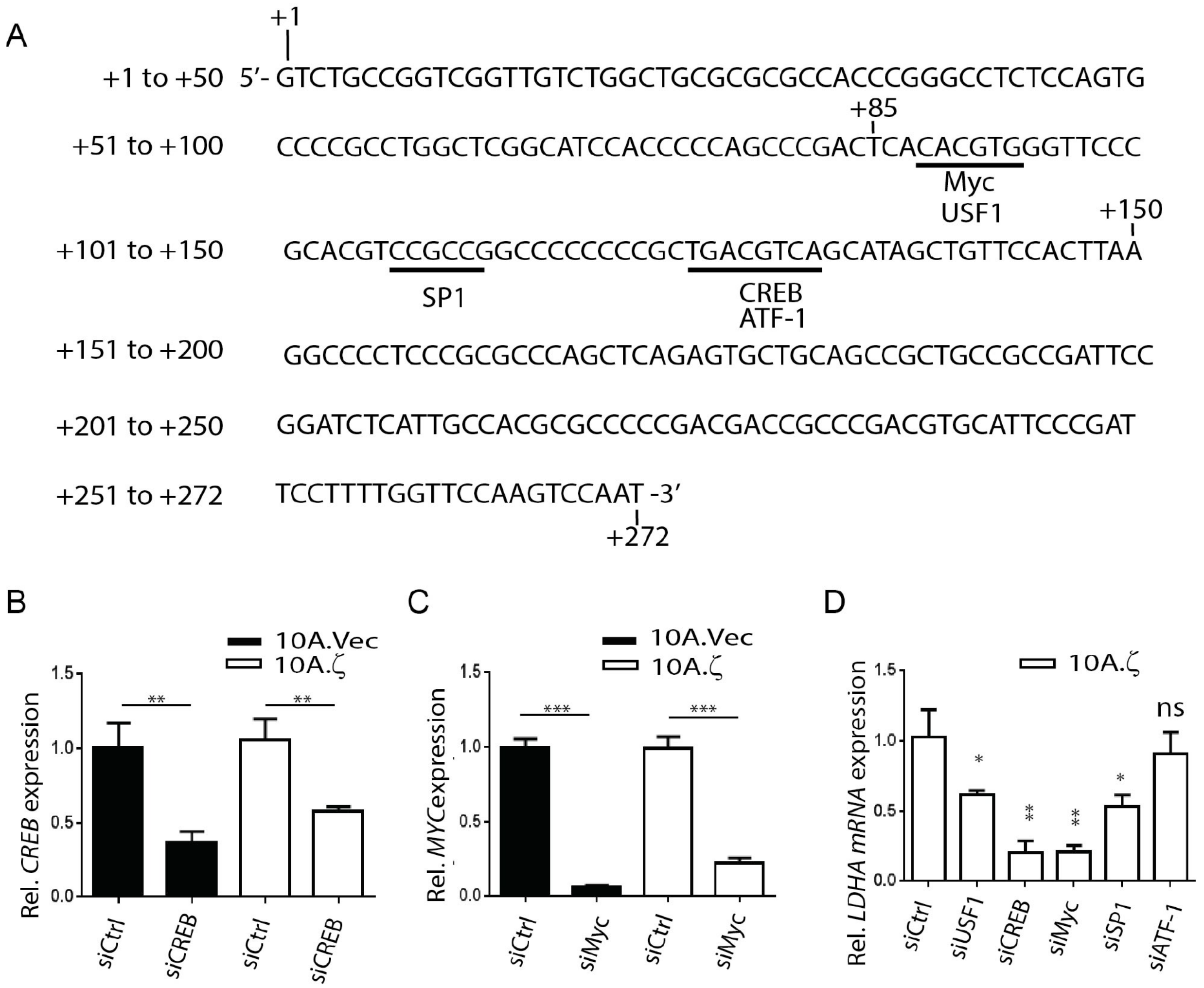

Supplementary Figure S4: Identification of cis-regulatory elements and transcription factors involved in LDHA transcriptional upregulation. **A.** Schematic overview of the transcription factor binding sites from +1 to +272 bp of LDHA's 5'-UTR annotated using the Transcription Element Search System (TESS). **B.** qRT-PCR analysis of relative *CREB* mRNA expression in the indicated cells was normalized by 18s mRNA expression. **C.** qRT-PCR analysis of relative *MYC* mRNA expression in the indicated cells was normalized by 18s mRNA expression. **D.** qRT-PCR analysis of relative *LDHA* mRNA expression in cells with five transcription factors individually knocked down and control cells. Bars indicate standard deviations. *, $P<0.05$; **, $P<0.01$; ***, $P<0.001$; n.s., not significant by the Student t-test.

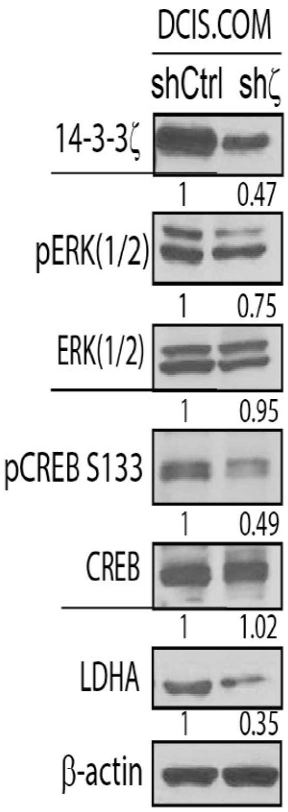

Supplementary Figure S5: DCIS.COM breast cancer cells. Western blotting of proteins in DCIS.COM sublines.

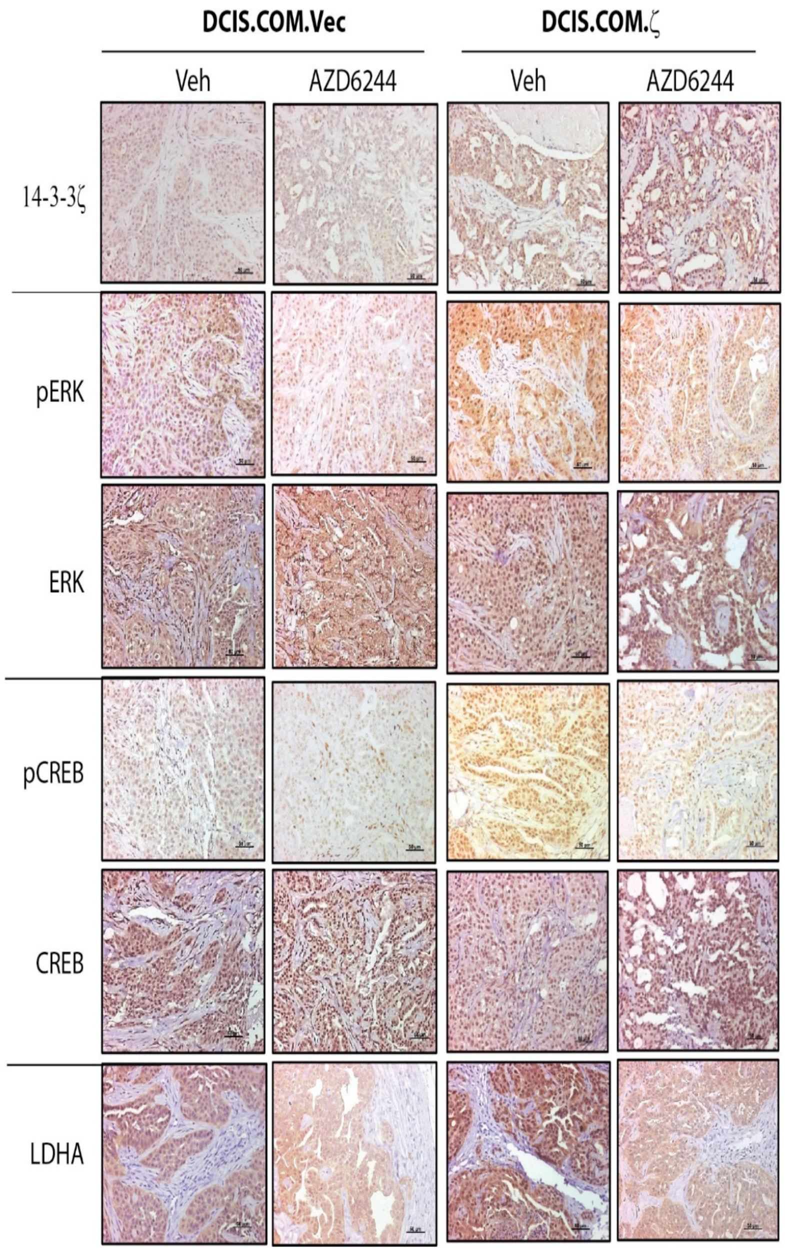

Supplementary Figure S6: Representative IHC staining of 14-3-3ζ, phospho-ERK, ERK, phospho-CREB, CREB, and LDHA in DCIS.COM.Vec and DCIS.COM.ζ tumors with indicated treatments. Quantitative analyses are shown in Supplementary Table 3. Scale bars, 50 µm.

Supplementary Table S1: Cellular glycolytic index of 14-3-3ζ overexpressing hMECs. The glycolytic activity of 14-3-3ζ overexpressing 10A.ζ and 12A.ζ cells was significantly increased than that of control 10A.Vec and 12A.Vec cells

Glycolytic Indices/hMEC lines	10A.Vec	10A.ζ	12A.Vec	12A.ζ
Glucose Uptake (G)	2.72	4.99	4.95	8.95
Lactate Production (L)	1.55	2.50	2.55	4.12
Oxygen Consumption (O)	2.97	1.80	2.44	1.32
Glycolytic Index (G x L / O)	1.42	6.93	5.17	27.93
Fold Change (Normalized by control)	1.00	4.88	1.00	5.40

Supplementary Table S2: Cellular glycolytic index of 14-3-3ζ-knockdown hMECs. The glycolytic activity of 14-3-3ζ-knockdown 10A.shζ and 12A.shζ cells was significantly reduced than that of control shRNA-transfected 10A.shCtrl and 12A.shCtrl cells

Glycolytic Indices/hMEC lines	10A.shCtrl	10A.shζ	12A.shCtrl	12A.shζ
Glucose Uptake (G)	2.79	1.46	1.69	1.08
Lactate Production (L)	1.48	0.80	0.99	0.47
Oxygen Consumption (O)	2.57	2.20	2.53	2.32
Glycolytic Index (G x L / O)	1.61	0.53	0.66	0.22
Fold Change (Normalized by control)	1.00	0.32	1.00	0.33

Supplementary Table S3: Glycolytic activity of 14-3-3ζ-overexpressing hMECs with LDHA knockdown. 10A.ζ and 12A.ζ cells transfected with LDHA shRNA (10A.ζ.shLDHA and 12A.ζ.shLDHA); or with control shRNA (10A.ζ.shCtrl and 12A.ζ.shCtrl). The glycolytic activity of hMECs cells was calculated by glycolytic index

Glycolytic Indices/hMEC lines	10A.ζ.shCtrl	10A.ζ.shLDHA	12A.ζ.shCtrl	12A.ζ.shLDHA
Glucose Uptake (G)	1.84	1.10	2.05	1.47
Lactate Production (L)	7.86	5.56	6.75	4.35
Oxygen Consumption (O)	1.20	1.40	1.40	1.56
Glycolytic Index (G x L / O)	12.05	4.37	9.88	4.02
Fold Change (Normalized by control)	1.00	0.36	1.00	0.42

Supplementary Table S4: Quantitative analyses of IHC staining of the ERK/CREB signaling pathways. The IHC staining scores for 14-3-3ζ, p-CREB, LDHA were defined as 0, 1+, 2+, 3+, which indicate different level of expression. The Ki-67 index was defined as the percentage of Ki-67 positive cells on slides and the TUNEL index was counted the TUNEL positive cells on slides. *P* value was calculated between vehicle and AZD6244 treated DCIS.COM.ζ tumors. * indicates *P*<0.05, ** indicates *P*<0.01 and * indicates *P*<0.001 by the Student t-test**

Marker/Xenografts	DCIS.COM.Vec	DCIS.COM.ζ		
	Vehicle	Vehicle	AZD6244	*P* Value
**14-3-3ζ**	1.6±0.245	2.6±0.245	2.2±0.200	*P*=0.2415
**pERK**	1.6±0.400	2.8±0.200	1.6±0.245	*P*=0.0053**
**pCREB**	2.4±0.245	3.0±0.000	2.2±0.200	*P*=0.0039**
**LDHA**	1.6±0.245	2.6±0.245	1.6±0.245	*P*=0.0203*
**Ki-67**	23%±4.359	37%±5.831	10%±2.739	*P*=0.0006***
**TUNEL**	4±1.673	3.2±0.583	4.4±1.631	*P*=0.508

Supplementary Table S5: TMA analyses. Analyses of 14-3-3ζ association with LDHA and pCREB (Ser-133) in consecutive TMA slides. A total of 187 (89%) specimens were examined the association between 14-3-3ζ and LDHA; and a total of 183 (88%) specimens were examined the association between 14-3-3ζ and phospho-CREB (Ser-133). The square of Pearson coefficient R^2 is 0.242 between 14-3-3ζ and LDHA; and R^2 is 0.245 between 14-3-3ζ and phospho-CREB. The IHC staining scores for 14-3-3ζ, p-CREB, LDHA were defined as 0, 1+, 2+, 3+, which indicate different level of expression. The square of Pearson coefficient R^2 were calculated and indicated the correlation relationship. *P* value was calculated by Chi-square analysis. * indicates *P*<0.05 and * indicates *P*<0.001 by Student t-test**

Marker	14-3-3ζ expression				*P* value	R^2	N
	0	1+	2+	3+			
LDHA expression							
0	4.2%	4.2%	5.3%	0.0%			
1+	5.3%	12.9%	10.6%	2.6%	0.047*	0.242	187
2+	6.4%	16.5%	9.6%	5.8%			
3+	1.6%	5.8%	3.7%	4.2%			
pCREB expression							
0	9.6%	5.5%	9.3%	0.5%			
1+	0.0%	9.8%	9.3%	4.4%	0.0006***	0.245	183
2+	0.0%	6.6%	9.8%	6.0%			
3+	3.8%	8.2%	12.0%	5.5%			
